# Association of air pollution and prior COVID-19 with atopic dermatitis risk: an interaction analysis in the UK biobank

**DOI:** 10.3389/fpubh.2026.1768057

**Published:** 2026-03-20

**Authors:** Yuanyi Guo, Dawei Zhou, Jiaqing Xiong

**Affiliations:** 1 The First Hospital of Hunan University of Chinese Medicine, Changsha, China; 2 The Third Xiangya Hospital, Central South University, Changsha, China

**Keywords:** air pollution, atopic dermatitis, COVID-19, epidemiology, public health

## Abstract

**Background and objective:**

Long-term air pollution is an established risk factor for atopic dermatitis (AD), but modifiers of this risk, particularly following post-COVID-19 immune alterations, are poorly understood. We investigated whether prior COVID-19 infection modifies the association between air pollution and incident AD.

**Methods:**

From an initial cohort of 502,357 UK Biobank (UKB) participants, a final analytic sample of 173,766 individuals was included after excluding those with missing data or indeterminate COVID-19 status. Incident AD was identified via ICD-10 code L20. Multivariable logistic regression was used to examine associations between long-term PM_2.5_, NO_2_, and NO_x_ exposure and AD risk, adjusting for age, sex, Townsend Deprivation Index, BMI, and lifestyle factors. The synergistic interaction between PM_2.5_ and COVID-19 was evaluated on both multiplicative and additive scales.

**Results:**

A significant association was identified between long-term PM_2.5_ exposure and a higher likelihood of AD (OR = 1.04, 95% CI 1.01–1.07). While prior COVID-19 infection was not an independent risk factor (OR = 0.98, 95% CI 0.91–1.05), it significantly modified the effect of PM_2.5_, with a synergistic interaction observed (*P* for interaction = 0.018). A synergistic interaction was observed, meaning the combined effect of air pollution and COVID-19 history exceeded the sum of their individual risks.

**Conclusion:**

Chronic PM_2.5_ exposure is linked to a heightened AD risk, which is then markedly amplified in individuals with a prior history of COVID-19 infection. Our findings suggest that prior viral infections can sensitize individuals to the dermatological effects of air pollution, defining a vulnerable subgroup that may benefit from targeted environmental health strategies.

## 1 Introduction

Atopic dermatitis (AD), as a common inflammatory skin disease, is marked by repeated relapses and severe itching, adversely affecting both the physical and mental health of individuals (1). The pathogenesis of AD is thought to be the result of a combination of genetic susceptibility (such as FLG gene mutations) and environmental factors (2, 3). A large body of epidemiological evidence indicates that air pollution is a major environmental factor for the incidence and exacerbation of AD (4). Fine particulate matter (PM_2.5_, PM_10_), nitrogen dioxide (NO_2_) and other ambient air pollutants are positively associated with the prevalence and severity of AD. Proposed mechanisms involve the induction of oxidative stress and skin barrier dysfunction (5–7). While air pollution is an independent risk factor, its heterogeneous effects across different sub-populations remain under-explored.

The Corona virus disease 2019 (COVID-19) pandemic has triggered complex immune disturbances alongside social behavioral changes (8, 9). Following infection, individuals often experience systemic immune dysregulation and heightened inflammatory responses (10, 11), which may potentially exacerbate the pathological processes of AD. A large cohort study showed an increased risk of AD after COVID-19 infection (12). Based on the preceding evidence, we pose the following research question: Does COVID-19 play a moderating or amplifying role in the skin-barrier damage and immune imbalance induced by air pollution? We hypothesize that these two factors may have a significant joint impact on the onset of AD. The biological plausibility of such a “two-hit” model is supported by evidence in other respiratory and systemic conditions, where air pollution has been found to exacerbate the pathogenic effects of common respiratory viruses. For instance, ambient fine particulate matter can the host’s susceptibility and inflammatory response to viruses such as influenza and respiratory syncytial virus (RSV) (13, 14). Furthermore, emerging research indicates that SARS-CoV-2 infection can induce prolonged immunological dysfunction, characterized by persistent cytokine elevation and systemic endothelial disturbances (15). This suggests a broader paradigm where prior viral insults may “prime” the immune system, potentially lowering the physiological threshold for damage induced by subsequent environmental stressors like PM_2.5_.

Existing literature has made progress in studying the individual impacts of air pollution and COVID-19 on AD (16, 17), but significant gaps remain: most studies have not systematically examined the interaction between these exposures, many are confined to small-scale regions and lack large-cohort data, and although inflammation-related responses have been explored, the specific roles of these pathways in AD pathogenesis remain unclear. To address these shortcomings, we will leverage the UK Biobank to execute a comprehensive, long-duration cohort study with multi-year follow-up of hundreds of thousands of participants. By systematically tracking air-pollution levels, COVID-19 exposure history, and AD incidence, we aim to assess the interaction between air pollution and COVID-19 on AD risk. This research seeks to provide evidence for identifying vulnerable subgroups and developing targeted prevention strategies.

## 2 Materials and methods

### 2.1 Study population

Data for this study were obtained from the UKB (No. 104784), a nationwide prospective cohort involving nearly 500,000 participants aged 40–69 years, enrolled across the UK between 2006 and 2010. Participants provided extensive information on their lifestyle and medical history, donated biological samples (blood, urine, and saliva) for future analysis, and consented to long-term health tracking via linkage to health-related records. We obtained electronic informed consent from all participants prior to their enrollment. The research protocol received ethical approval from both the National Health Service (NHS) and the National Research Ethics Service (NRES).

### 2.2 Ascertainment of AD

AD diagnosis was established using participant self-reports and medical records from inpatient or primary care. The diagnosis of AD was recorded using the International classification of diseases (ICD) coding system (ICD-10 code: L20). The participants were followed up starting from the baseline until they were first diagnosed with AD, died, were lost to follow-up, or until August 2024. After comprehensively considering the years of air pollution data and the definition of COVID-19 susceptibility, only AD data after 2000 were used for the regression analysis.

### 2.3 Estimation of exposure to air pollution

The levels of exposure to air pollutants were estimated at baseline. Air pollutants in this study included NO_x,_ NO_2,_ PM_2.5_ and PM_10_. A land use regression (LUR) model was constructed to calculate the concentrations of PM_2.5_, PM_2.5–10_, PM_10_, NO_x_, and NO_2_ (18). Air pollution measurements were conducted between October 2008 and April 2011. LUR models are based on the intensity of a range of variables such as traffic intensity, land use, and topography and allow for the estimation of spatial variation in air pollutant concentrations at residential addresses provided by participants at baseline (19). For the analysis, each pollutant’s concentration was categorized into low, medium, and high exposure levels based on quartiles. The specific concentration thresholds for each category are detailed in Supplementary Table S2. Due to high correlation and shared missing data patterns, pollutants were grouped into gaseous pollutants (NO_x_/NO_2_) and particulate matter (PM_10_/PM_2.5_) for subsequent regression analyses.

### 2.4 COVID-19 susceptibility grouping

**Figure 1:**
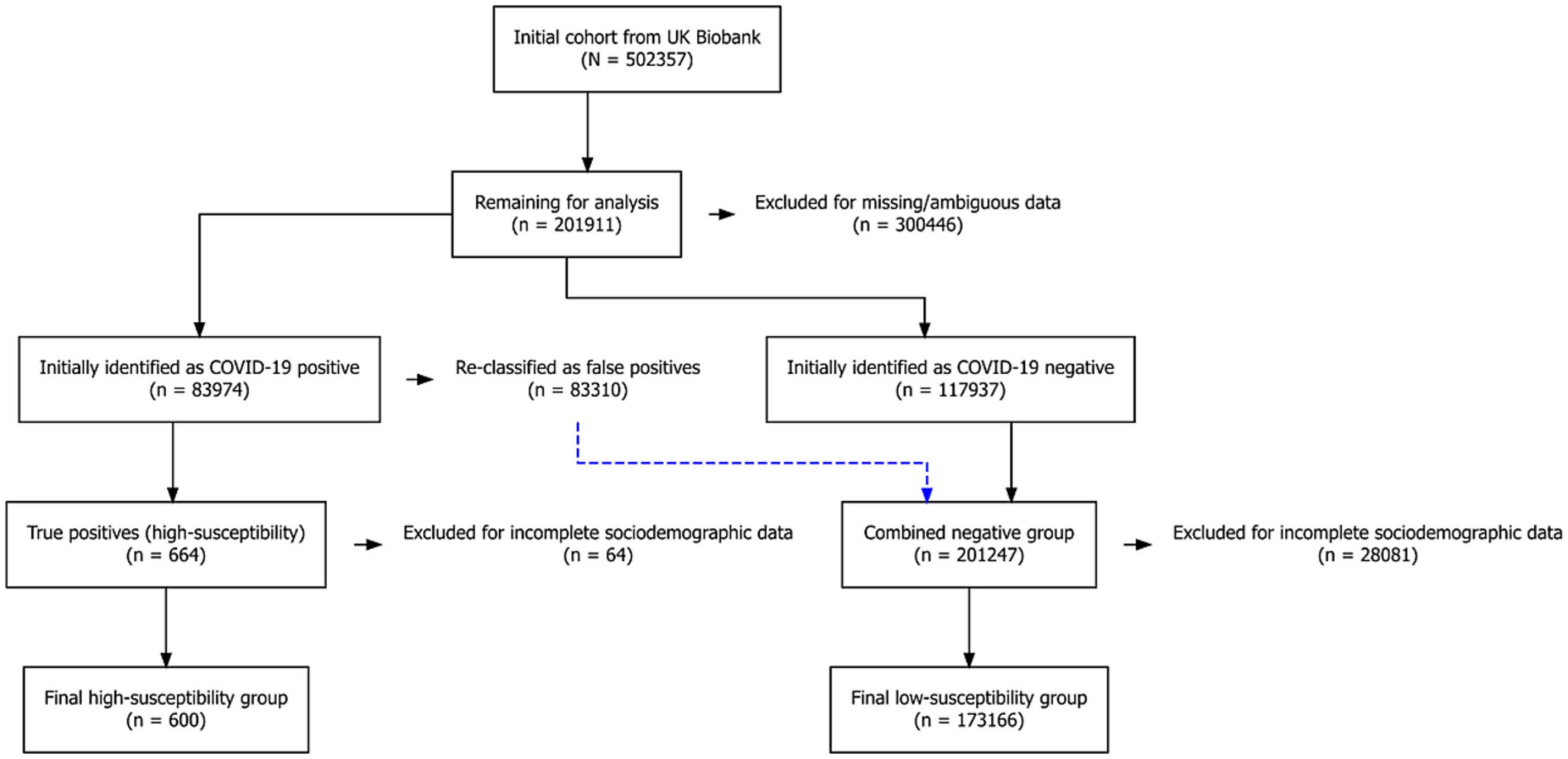
Flow diagram of cohort selection and stratification.

Considering that directly evaluating an individual’s susceptibility to COVID-19 is relatively complex, this study used whether an individual has ever had COVID-19 as a surrogate indicator for infection history. Based on the COVID-19 testing records in the UKB, participants were divided into two susceptibility groups. The high-susceptibility group includes any individual with at least one positive COVID-19 test result, regardless of infection timing or symptom severity, under the assumption that a documented infection reflects a relatively greater likelihood of becoming infected when exposed. The low-susceptibility group comprises individuals whose test records are consistently negative, suggesting a comparatively lower susceptibility. Although infection status does not capture all factors influencing susceptibility, this dichotomous classification provides a feasible basis for examining the association between COVID-19 susceptibility and the risk of developing AD under the current data constraints. The detailed selection flow is illustrated in Figure 1.

The analysis began with 502,357 participants from the UKB. Individuals with missing key variables were excluded (n = 300,446, 59.8% of the initial sample). To minimise the risk of false-positive COVID-19 classification, a further 83,310 participants were removed (16.6% of the original cohort). After additionally excluding participants with incomplete sociodemographic information, the final analytic cohort comprised 173,766 individuals, including 600 confirmed COVID-19 cases and 173,166 COVID-negative controls. The substantial sample attrition was primarily due to missing environmental data and the subset-specific nature of COVID-19 testing in the UKB. A comparison between included and excluded participants (Supplementary Table S1) revealed that the final analytic cohort remains representative of the overall UKB population (all SMD ≤ 0.25).

### 2.5 Covariates

We intended to include age, gender, townsend deprivation index (TDI), body mass index (BMI), education score, income score and smoking status as covariates to exclude the interference of confounding factors on the results. Age was calculated using the date of birth and baseline assessment date. Used the registered gender. The TDI was calculated based on percentages of unemployment, overcrowding, lack of resources, and lack of housing insecurity and could be downloaded from UKB. Obesity was defined as a BMI ≥ 28 kg/m^2^. This threshold was selected based on its higher sensitivity in detecting adiposity-related inflammatory risk in middle-aged and older European populations, where standard cutoffs may underestimate systemic inflammation. Both the education score and the income score used the England points system. Smoking status was determined as ever and never.

### 2.6 Statistical analysis

We aimed to explore the link between air pollution, COVID-19 infection, and the onset of AD. The analyses comprised means, medians, standard deviations, and ranges. Summary statistics were used for continuous data, whereas categorical variables were described by frequencies. The distribution of data was assessed using normality tests to determine the appropriate statistical approach for further analysis. For comparisons between categorical data groups, when the expected frequency was less than or equal to 5, we used Fisher’s exact test; otherwise, we used the chi-square test. We employed univariable logistic regression to identify factors (NO_x_, NO_2_, PM_10_, and PM_2.5_) associated with incident AD risk. This step helps identify factors that may be associated with the outcome variable. Subsequently, multivariable logistic regression models were used to determine independent predictors while adjusting for potential confounders. We adopted a theory-driven approach by incorporating all pre-specified covariates (age, sex, BMI, TDI, ethnicity, education, and smoking status) into the multivariable models, regardless of their univariate significance. This strategy ensures robust adjustment for known confounders and minimizes residual confounding. Statistical analyses were distributed as follows: SPSS (version 27) for baseline comparisons; R (version 4.2.2) for multivariable modeling, interaction analysis, and figure generation; and MSTATA for independent data verification.

## 3 Results

### 3.1 Characteristics of the study cohort by atopic dermatitis (AD) status

Of the total data of 502,357 people, 427,335 people with complete data were finally included. 10,138 (2.4%) developed incident AD during the follow-up period. Table 1 summarized the baseline characteristics of the cohort, with participants grouped by their AD status. The baseline characteristics of participants stratified by their COVID-19 infection status were also compared. We observed significant differences between the COVID-19 positive and negative groups in terms of age, sex, socioeconomic status, and ethnic background. A detailed comparison is provided in Supplementary Table S1.

**Table 1:** Comparison of baseline characteristics by atopic dermatitis (AD) incidence status in the UK biobank cohort.

Characteristic	AD		*p*-value^2^
	No AD, *N* = 417,197^1^	Incident AD, *N* = 10,138^1^	
Age	57 ± 8	57 ± 8	**0.024**
Sex			**<0.001**
Female	226,844 (54.4%)	5,887 (58.1%)	
Male	190,353 (45.6%)	4,251 (41.9%)	
BMI^3^	26.7 (24.1, 29.9)	26.8 (24.1, 30.0)	0.431
TDI^3^	−1.34 ± 3.03	−1.35 ± 2.99	0.779
Education score	10 (4, 21)	10 (4, 22)	**0.002**
Ethnic background			**<0.001**
Asian-related	9,895 (2.4%)	336 (3.3%)	
Black and related	7,261 (1.7%)	137 (1.4%)	
British	368,620 (88.4%)	8,950 (88.3%)	
Others	4,879 (1.2%)	90 (0.9%)	
White and mixed white-related	26,542 (6.4%)	625 (6.2%)	
Smoking status			0.312
No	11,599 (60.1%)	281 (57.8%)	
Yes	7,702 (39.9%)	205 (42.2%)	
Unknown	397,896	9,652	

^1^Continuous data as mean ± standard deviation (SD), categorical data as number (percentage), and median with inter quartile range (IQR).

^2^Welch Two Sample *t*-test; Pearson’s Chi-squared test; Wilcoxon rank sum test.

^3^BMI, Body Mass Index; TDI, Townsend Deprivation Index. Bold values indicate statistical significance (*p* < 0.05).

Compared to the non-AD group (*N* = 417,197), participants in the incident AD group (*N* = 10,138) were slightly older (mean age 57 ± 8 years for both groups; *p* = 0.024) and exhibited a greater percentage of females (58.1% vs. 54.4%; *p* < 0.001). Notable disparities were also observed in education score (*p* = 0.002) and ethnic background (*p* < 0.001). Specifically, the proportion of individuals with an Asian-related background was higher in the AD group (3.3% vs. 2.4%). Statistical analysis revealed no significant variations when comparing BMI (*p* = 0.431), TDI (*p* = 0.779), and smoking status (*p* = 0.312) across the two cohorts.

### 3.2 The effect of air pollution on the onset of AD

Table 2 presented the results of the univariate association analysis between air pollutant exposure levels and the risk of incident AD.

**Table 2:** Univariate association of air pollutant exposure with incident atopic dermatitis (AD) risk.

Characteristic^1^	AD		*p*-value^2^	OR^3^	95% CI^3^
	No AD	Incident AD			
NO_2_					
Low level	105,342	1,441		Ref	Ref
Medium level	210,264	3,555	**<0.001**	1.24	1.17, 1.32
High level	103,631	1,693	**<0.001**	1.20	1.12, 1.29
NO_x_					
Low level	105,804	1,438		Ref	Ref
Medium level	211,419	3,560	**<0.001**	1.24	1.17, 1.32
High level	102,014	1,691	**<0.001**	1.22	1.14, 1.31
PM_10_					
Low level	103,117	1,560		Ref	Ref
Medium level	214,369	3,433	0.060	1.06	1.00, 1.13
High level	101,516	1,695	**0.005**	1.11	1.03, 1.18
PM_2.5_					
Low level	104,460	1,477		Ref	Ref
Medium level	213,771	3,519	**<0.001**	1.17	1.10, 1.24
High level	100,771	1,692	**<0.001**	1.19	1.11, 1.28

^1^Characteristic: Air pollutant exposure level. Data for NO_2_/NO_x_ are identical, and data for PM_2.5_/PM_10_ are identical. Exposure levels are defined as follows:

NO_2_: Low (≤21.415 ug/m^3^), Medium (21.415–31.315 ug/m^3^), and High (>31.315 ug/m^3^).

NOx: Low (≤34.290 ug/m^3^), Medium (34.290–50.995 ug/m^3^), and High (>50.995 ug/m^3^).

PM10: Low (≤15.225 ug/m^3^), Medium (15.225–17.045 ug/m^3^), and High (>17.045 ug/m^3^).

PM2.5: Low (≤9.280 ug/m^3^), Medium (9.280–10.580 ug/m^3^), and High (>10.580 ug/m^3^).

^2^Pearson’s chi-squared test was utilized to assess the association with AD status.

^3^OR: Odds Ratio; CI: Confidence Interval. Bold values indicate statistical significance (*p* < 0.05).

Overall, the analysis revealed that heightened exposure to most examined air pollutants was significantly correlated with an elevated risk of incident AD, generally demonstrating a graded increase in odds ratios as exposure levels rose from low to medium and high.

An increased risk of incident AD was significantly associated with both medium and high exposure levels of NO_2_, compared to the low-level reference group. For high exposure, the OR was 1.20 (95% CI: 1.12–1.29, *p* < 0.001). Likewise, a strong positive correlation was identified between NO_x_ exposure and the onset of AD. Both medium-level and high-level exposure were identified as significant predictors, with corresponding OR of 1.24 (95% CI: 1.17–1.32, *p* < 0.001) and 1.22 (95% CI: 1.14–1.31, *p* < 0.001), respectively.

In the case of PM_10_, only the highest exposure tier exhibited a statistically significant association with incident AD (OR = 1.11, 95% CI: 1.03–1.18, *p* = 0.005). While PM_10_ at medium exposure levels showed a borderline increase in risk (OR = 1.06, 95% CI: 1.00–1.13), the *p*-value of 0.060 indicated that this finding was not statistically significant. However, PM_2.5_ demonstrated a strong dose–response relationship with AD, as both medium (OR = 1.17, 95% CI: 1.10–1.24, *p* < 0.001) and high (OR = 1.19, 95% CI: 1.11–1.28, *p* < 0.001) exposure levels were independently associated with a significantly higher risk of AD. These univariate findings collectively indicate that higher concentrations of common air pollutants, particularly NO_2_, NO_x_, and PM_2.5_, are independently associated with an increased risk of developing AD.

**Figure 2:**
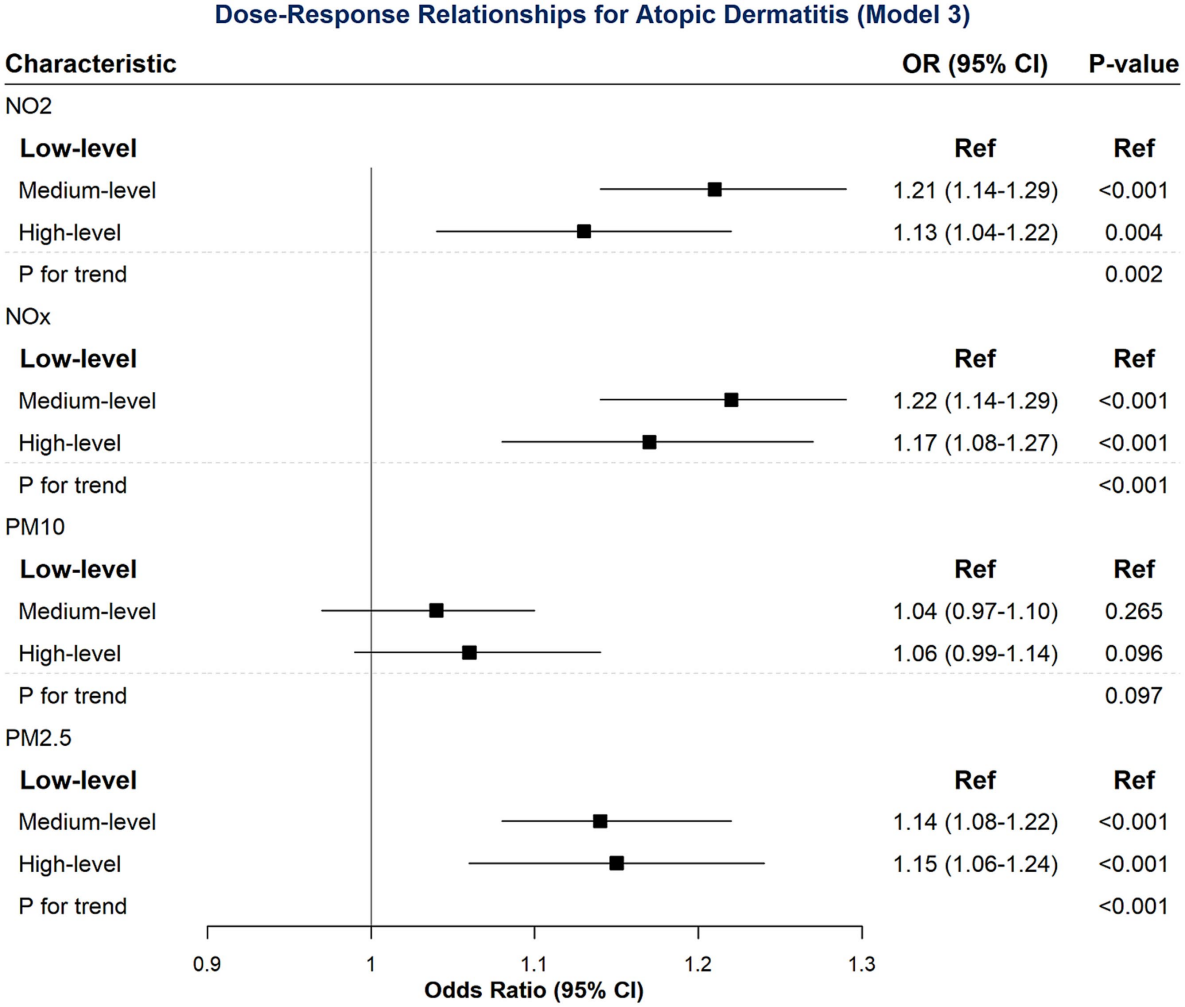
Dose–response relationships for atopic dermatitis (Model 3).

Table 3 presented the results from three hierarchical adjustment models. To further elaborate on the findings of the most comprehensively adjusted model (Model 3), Figure 2 provided a visual representation as a forest plot, allowing for an intuitive comparison of the OR and their 95% CI across different pollutants and exposure levels.

**Table 3:** Dose–response relationships between air pollutants and atopic dermatitis incidence across hierarchical adjustment models.

Characteristic	Model 1^2^			Model 2^2^			Model 3^2^		
	OR^1^	95% CI^1^	*p*-value	OR^1^	95% CI^1^	*p*-value	OR^1^	95% CI^1^	*p*-value
NO_2_									
Low level	Ref	Ref		Ref	Ref		Ref	Ref	
Medium level	1.24	1.17, 1.32	<0.001	1.23	1.16, 1.31	<0.001	1.21	1.14, 1.29	**<0.001**
High level	1.20	1.12, 1.29	<0.001	1.18	1.09, 1.26	<0.001	1.13	1.04, 1.22	**0.004**
*P* for trend			<0.001			<0.001			**0.002**
NO_x_									
Low level	Ref	Ref		Ref	Ref		Ref	Ref	
Medium level	1.24	1.17, 1.32	<0.001	1.23	1.16, 1.31	<0.001	1.22	1.14, 1.29	**<0.001**
High level	1.22	1.14, 1.31	<0.001	1.21	1.13, 1.30	<0.001	1.17	1.08, 1.27	**<0.001**
*P* for trend			<0.001			<0.001			**<0.001**
PM_10_									
Low level	Ref	Ref		Ref	Ref		Ref	Ref	
Medium level	1.06	1.00, 1.13	0.060	1.05	0.99, 1.12	0.115	1.04	0.97, 1.10	0.265
High level	1.11	1.03, 1.18	0.005	1.09	1.01, 1.17	0.018	1.06	0.99, 1.14	0.096
*P* for trend			0.005			0.018			0.097
PM_2.5_									
Low level	Ref	Ref		Ref	Ref		Ref	Ref	
Medium level	1.17	1.10, 1.24	<0.001	1.16	1.09, 1.23	<0.001	1.14	1.08, 1.22	**<0.001**
High level	1.19	1.11, 1.28	<0.001	1.19	1.11, 1.27	<0.001	1.15	1.06, 1.24	**<0.001**
*P* for trend			<0.001			<0.001			**<0.001**

^1^OR, Odds Ratio, CI, Confidence Interval.

^2^Model 1: no covariates were adjusted; Model 2: adjusted for age, sex, and Ethinc; Model 3: adjusted for age, sex, Ethinc, BMI, Townsend, and educate. Bold values indicate statistical significance (*p* < 0.05).

Table 3 further examined the dose–response relationships between air pollutant exposure and incident AD after hierarchical adjustments. Focusing on Model 3, the most comprehensively adjusted model, key findings emerged.

Consistent with Table 2, NO_2_ and NO_x_ exposure maintained significant associations with increased AD risk across medium and high levels, showing persistent dose–response trends. In contrast, PM_10_ and AD risk, which showed some significance in Table 2, became non-significant in Model 3. Conversely, PM_2.5_ continued to demonstrate robust and significant associations with AD incidence in Model 3 (medium: OR = 1.14, *p* < 0.001; high: OR = 1.15, *p* < 0.001), consistent with Table 2, and maintained a strong dose–response trend (*P* for trend < 0.001).

In summary, after comprehensive adjustment, NO_2_, NO_x_, and PM_2.5_ exposure consistently demonstrated a dose-dependent increase in AD risk, while the initial link with PM_10_ did not persist.

### 3.3 The effect of COVID-19 susceptibility on the onset of AD

The crude incidence of AD was slightly lower in the COVID-19 positive group (1.8%) compared to the negative group (2.3%), though this difference was not statistically significant (Supplementary Table S3).

To further investigate this association, we performed logistic regression analysis (Table 4). The unadjusted univariate model (Model 1) indicated that COVID-19 infection was not a significant predictor of AD development (OR = 0.78, 95% CI: 0.43–1.42, *p* = 0.418). This lack of significance remained even after adjusting for covariates such as age, sex, BMI, TDI, and ethnicity in the multivariate model (Model 2) (OR = 0.78, 95% CI: 0.43–1.42, *p* = 0.424).

**Table 4:** Logistic regression results for univariate and multivariate models assessing risk of incident atopic dermatitis.

Characteristic	Model 1^1^			Model 2^2^		
	OR^3^	95% CI	*p*-value	OR	95% CI	*p*-value
COVID-19 Status						
Negative	Ref			Ref		
Positive	0.78	0.43, 1.42	0.418	0.78	0.43, 1.43	0.424
Age	–			1.00	0.99, 1.00	0.915
Sex						
Female	–			Ref		
Male	–			1.14	1.07, 1.22	**<0.001**
BMI	–			1.00	0.99, 1.01	0.211
TDI	–			1.00	0.99, 1.01	0.967
Ethnic						
British	–			Ref		
Asian-related	–			1.40	1.05, 1.85	**0.021**
Black and related	–			0.88	0.57, 1.35	0.553
Others	–			1.09	0.96, 1.25	0.189
White-related	–			0.92	0.61, 1.39	0.689
Education score	–			0.99	0.99, 1.00	0.621

^1^Model 1 represents the unadjusted univariate logistic regression for the primary exposure (COVID-19 status). Covariates were not included in this model, indicated by “–”.

^2^Model 2 represents the multivariate logistic regression, adjusted for all variables listed in the table: COVID-19 status, age, sex, BMI, Townsend Deprivation Index, education score, and ethnic background.

^3^OR, Odds Ratio; BMI, Body Mass Index; CI, Confidence Interval; Ref., Reference group. Bold values indicate statistical significance (*p* < 0.05).

### 3.4 The combined effect of air pollution and COVID-19 susceptibility on the risk of AD onset

We next explored whether the effect of air pollutants on AD risk was modified by COVID-19 infection status using multivariate logistic regression models. The results are presented in Table 5.

**Table 5:** Interaction between air pollutants and COVID-19 status on the risk of incident atopic dermatitis.

Term	Model 1 (NO_2_)^1^	Model 2 (NO_x_)	Model 3 (PM_10_)	Model 4 (PM_2.5_)
	OR (95% CI)^4^	OR (95% CI)	OR (95% CI)	OR (95% CI)
Main effects^2^				
COVID-19 (Positive vs. Negative)	1.26 (0.69–2.29)	–	–	–
Air pollutant	1.00 (0.99–1.01)	1.00 (0.99–1.00)	1.01 (0.99–1.03)	**1.04 (1.01–1.07)**
Interaction term				
COVID-19 * Air Pollutant	1.00 (0.99–1.01)	1.00 (1.00–1.00)	1.01 (0.99–1.03)	**1.03 (1.01–1.06)**
*p*-value for interaction^3^	0.568	0.091	0.094	**0.018**

^1^Each model was run separately for one air pollutant and its interaction with COVID-19 status. All models were adjusted for age, sex, BMI, Townsend Deprivation Index, education score, and ethnic background.

^2^The main effect of COVID-19 is shown for the NO_2_ model as an example and was similar across models.

^3^The *p*-value for interaction tests the hypothesis that the effect of the air pollutant on AD risk is different between COVID-19 positive and negative groups.

^4^OR, Odds Ratio; CI, Confidence Interval. Bold values indicate statistical significance (*p* < 0.05).

A meaningful statistical interaction was observed between PM_2.5_ exposure and COVID-19 status (*P* for interaction = 0.018). Within this model, the main effect of PM_2.5_ was additionally linked to a heightened AD risk (OR = 1.04, 95% CI: 1.01–1.07). The significant interaction term (OR = 1.03, 95% CI: 1.01–1.06) suggested a statistical synergistic interaction, indicating that the adverse impact of PM_2.5_ on AD risk appeared to be amplified in individuals with a prior COVID-19 infection. However, given the relatively small number of AD cases within the COVID-19 positive subgroup (*N* = 11), these findings should be interpreted as exploratory rather than confirmatory. Further large-scale studies are needed to evaluate the stability of these interaction estimates. In contrast, no significant interactions were found for NO_2_, NO_x_, or PM_10_, although the interactions for NO_x_ and PM_10_ approached statistical significance (*p* = 0.091 and *p* = 0.094, respectively).

## 4 Discussion

Within this extensive prospective cohort study, we elucidated the complex interplay between air pollution, viral infection history, and the risk of incident AD. Our principal findings are threefold: first, we confirmed that long-term exposure to traffic-related pollutants, particularly NO_2_, NO_x_, and PM_2.5_, is an independent, dose-dependent risk factor for adult-onset AD. Second, a prior COVID-19 infection, when analyzed alone, was not significantly associated with AD risk. However, we identified a statistically significant synergistic interaction between PM_2.5_ and COVID-19 history. This suggests that a history of COVID-19 may act as a potential sensitizing factor that modifies the body’s response to environmental stressors, although this finding remains preliminary given the sample constraints.

Our findings on air pollution align with and extend the evidence cited in our introduction. The robust association with NO_2_, NO_x_, and PM_2.5_, even after comprehensive adjustment, reinforces the role of these pollutants in skin barrier disruption and inflammation (20). An intriguing aspect of our results was the divergent association for PM_2.5_ and PM_10_. The persistence of the PM_2.5_ effect, contrasted with the attenuation of the PM_10_ effect after adjustment, is biologically plausible. Due to their smaller size and larger surface area for carrying toxic adsorbents like polycyclic aromatic hydrocarbons (PAHs), PM_2.5_ particles can penetrate the epidermis more effectively, inducing potent oxidative stress via pathways like the aryl hydrocarbon receptor (AhR), and promoting a Th2-skewed immune response characteristic of AD (21, 22). The loss of association for PM_10_ likely indicates that its initial correlation was confounded by co-exposure to more pathogenic fine-fraction pollutants.

The most novel, albeit exploratory, finding of our study is the statistical interaction between COVID-19 and PM_2.5_. While a history of COVID-19 alone did not increase AD risk—a finding that contrasts with some reports (12) but must be interpreted cautiously due to the low number of events in our cohort—its role as an effect modifier was clear. We propose a “dual-insult” hypothesis to explain this interaction. The first insult, COVID-19 infection, can induce a systemic, pro-inflammatory state that may persist subclinically long after viral clearance, a phenomenon recognized in “Long COVID” (23). This systemic inflammation, potentially involving widespread endothelial cell damage, may lead to an “inside-out” compromise of skin barrier homeostasis, creating a state of heightened vulnerability (24). The second insult is the chronic “outside-in” assault from PM_2.5_. When a pre-sensitized skin barrier is subjected to the potent oxidative and inflammatory stress from PM_2.5_, the cumulative damage may surpass the threshold for clinical disease manifestation. The biological plausibility of this model is further supported by studies on other respiratory viruses, such as influenza and respiratory syncytial virus (RSV), where air pollutants have been shown to exacerbate viral-induced inflammation and vice versa. We emphasize that this “two-hit” model is currently a mechanistic hypothesis derived from statistical observations. Given that only 11 AD events occurred in the COVID-19 positive group, the stability of this interaction estimate must be interpreted with caution, and a direct biological causal link remains to be proven.

Several methodological choices warrant discussion. Regarding the use of a BMI threshold of ≥28 kg/m^2^ in a UK-based cohort, we believe this is justified by the age demographic of the UKB (40–69 years). Recent evidence from European aging cohorts suggests that the standard 30 kg/m^2^ cutoff may underestimate adiposity-related inflammatory risks in middle-aged and older adults due to age-related shifts in body composition (e.g., sarcopenic obesity). Research indicates that thresholds of 27–28 kg/m^2^ provide higher sensitivity for identifying systemic inflammation in these populations (25, 26). Furthermore, the lack of a significant independent effect of BMI in our models (*p* = 0.211) suggests our primary findings are robust to this classification.

This study has notable strengths, as outlined in our introduction, including its prospective design, large and well-characterized cohort, and use of high-resolution exposure models. However, several limitations must be acknowledged. First, the classification of COVID-19 status relied on documented positive tests. This may lead to exposure misclassification, as asymptomatic or untested individuals in the control group might have had prior infections. Such misclassification typically biases results toward the null, suggesting the observed interaction might be conservative. Second, the substantial sample attrition (from 502,357 to 173,766) could introduce selection bias. However, our sensitivity analysis showed that the included and excluded populations were highly comparable across key demographics (all SMDs ≤ 0.25), indicating that the analytic cohort remains representative of the UKB population. Finally, the small number of AD cases in the COVID-positive group (*N* = 11) limits our statistical power. Consequently, our interaction findings are preliminary and require validation in larger post-pandemic datasets.

In conclusion, our study provides the first epidemiological evidence suggesting that a history of COVID-19 infection may amplify the risk of AD associated with PM_2.5_ exposure. This highlights a potential new dimension of post-pandemic public health, identifying a vulnerable population subgroup that may require enhanced protection from air pollution. These findings warrant urgent validation in independent populations. Future research should focus on elucidating the underlying biological mechanisms through experimental studies. Furthermore, it is crucial to investigate whether this sensitizing phenomenon is specific to COVID-19 or represents a broader paradigm of post-viral susceptibility. Examining the interaction between air pollution and other major respiratory viruses, such as influenza and respiratory syncytial virus (RSV), which are also known to trigger distinct systemic immune responses and exacerbate allergic diseases, would be a critical next step in understanding the complex environmental and infectious triggers of chronic inflammatory diseases (27).

## 5 Conclusion

In this large-scale cohort, we confirmed chronic PM_2.5_ exposure as an independent risk factor for AD. Crucially, we identified a significant interaction where the adverse impact of PM_2.5_ was substantially amplified in individuals with a history of COVID-19 infection. These findings suggest that viral infections may potentially sensitize individuals to the effects of environmental stressors. However, due to the observational nature of this study and the limited number of AD events within the COVID-19 subgroup, these interaction findings remain preliminary and require confirmation in independent, larger cohorts. Our findings highlight a high-risk subgroup that could benefit from targeted public health strategies aimed at reducing air pollution exposure, warranting further mechanistic investigation.

## Data Availability

The datasets presented in this study can be found in online repositories. The names of the repository/repositories and accession number(s) can be found at: https://www.ukbiobank.ac.uk/.
