# Correction: Association of air pollution and prior COVID-19 with atopic dermatitis risk: an interaction analysis in the UK biobank

**DOI:** 10.3389/fpubh.2026.1854712

**Published:** 2026-04-20

**Authors:** Frontiers Production Office

**Affiliations:** Frontiers Media SA, Lausanne, Switzerland

**Keywords:** air pollution, atopic dermatitis, COVID-19, epidemiology, public health

Affiliation “The First Hospital of Hunan University of Chinese Medicine, Changsha, China” was erroneously given as “School of Stomatology, Hunan University of Chinese Medicine, Changsha, China”.

The present address has been removed for author Yuanyi Guo.

The original version of this article has been updated.

