# Supplementary material for: Association of air pollution and prior COVID-19 with atopic dermatitis risk: an interaction analysis in the UK biobank
Source: Front Public Health. 2026 Mar 20;14:1768057. doi: 10.3389/fpubh.2026.1768057 (PMC13047125; doi:10.3389/fpubh.2026.1768057)
Supplement: Supplementary file 1 [file supplementary_file_1.docx]

Supplementary Material

Table S1 Demographic and Sociodemographic Characteristics Stratified by COVID-19 Infection Status in the UK Biobank Cohort

| **Characteristic** | **COVID-19** | | | ***P*-value^2^** |
| --- | --- | --- | --- | --- |
|  | **Overall, N = 173,766** | **Negative, N = 173,166** | **Positive, N = 600^1^** |  |
| **Age** | 56 ± 8 | 56 ± 8 | 48 ± 6 | **< 0.001** |
| **Sex** |  |  |  | **0.004** |
| Male | 74,765 (43.0%) | 74,542 (43.0%) | 223 (37.2%) |  |
| Female | 99,001 (57.0%) | 98,624 (57.0%) | 377 (62.8%) |  |
| **BMI^3^** | 26.1 (23.7, 29.1) | 26.1 (23.7, 29.1) | 25.6 (23.1, 29.0) | **0.009** |
| **TDI** | -1.79 ± 2.75 | -1.79 ± 2.75 | -1.28 ± 3.03 | **< 0.001** |
| **Education Score** | 8 (3, 17) | 8 (3, 17) | 11 (5, 22) | **< 0.001** |
| **Ethinc Background** |  |  |  |  |
| British | 158,293 (91.1%) | 157,776 (91.1%) | 517 (86.2%) |  |
| Asian - related | 2,184 (1.3%) | 2,173 (1.3%) | 11 (1.8%) |  |
| White and Mixed White - related | 10,770 (6.2%) | 10,717 (6.2%) | 53 (8.8%) |  |
| Black and Related | 1,208 (0.7%) | 1,196 (0.7%) | 12 (2.0%) |  |
| Others | 1,311 (0.8%) | 1,304 (0.8%) | 7 (1.2%) |  |
| **Smoking Status** |  |  |  | 0.172 |
| No | 8,188 (61.5%) | 8,144 (61.5%) | 44 (69.8%) |  |
| Yes | 5,127 (38.5%) | 5,108 (38.5%) | 19 (30.2%) |  |
| Unknown | 160,451 | 159,914 | 537 |  |

^1^Data are presented as mean ± standard deviation (SD), number (n) with percentage, or median with inter quartile range (IQR)

^2^Welch Two Sample t-test; Pearson's Chi-squared test; Wilcoxon rank sum test

^3^ BMI, Body Mass Index; TDI, Townsend Deprivation Index

Table S2 Distribution of Air Pollutant Levels and Their Proportions

| **Air Pollutant Category** | **Air Pollutant Level^1^** | **Count** | **Percentage** |
| --- | --- | --- | --- |
| NO_2_ | Low level | 122,098 | 24.7% |
|  | Medium level | 250,412 | 50.6% |
|  | High level | 122,471 | 24.7% |
| NO_x_ | Low level | 122,305 | 24.7% |
|  | Medium level | 251,307 | 50.8% |
|  | High level | 121,369 | 24.5% |
| PM_10_ | Low level | 112,844 | 24.5% |
|  | Medium level | 235,178 | 51.0% |
|  | High level | 113,040 | 24.5% |
| PM_2.5_ | Low level | 114,381 | 24.8% |
|  | Medium level | 234,128 | 50.8% |
|  | High level | 112,553 | 24.4% |

^1^Exposure levels are defined as follows:

NO2: Low (<= 21.415 ug/m^3^), Medium (21.415~31.315 ug/m^3^), and High (> 31.315 ug/m^3^);.

NOx: Low (<= 34.290 ug/m^3^), Medium (34.290~50.995 ug/m^3^), and High (> 50.995 ug/m^3^).

PM10: Low (<= 15.225 ug/m^3^), Medium (15.225~17.045 ug/m^3^), and High (> 17.045 ug/m^3^).

PM2.5: Low (<= 9.280 ug/m^3^), Medium (9.280~10.580 ug/m^3^), and High (> 10.580 ug/m^3^).

Table S3 Cross-tabulation of COVID-19 Infection Status and Incident Atopic Dermatitis (AD)

| **COVID-19 Status** | **AD^1^** | | **Total** |
| --- | --- | --- | --- |
|  | **No AD, N = 169712** | **Incident AD, N = 173766** |  |
| Negative | 169123 (97.7%) | 4043 (2.3%) | 173166 (100%) |
| Positive | 589 (98.2%) | 11 (1.8%) | 600 (100%) |

^1^Data are presented as n (row %). P-value for the association was 0.417, derived from Pearson's Chi-squared test.
